# Discriminant study of cervical squamous cells based on computer image analysis

**DOI:** 10.1097/MD.0000000000045630

**Published:** 2025-11-14

**Authors:** Haiyan Niu, Jing Zheng, Na Xie, Chaona Jiang, Yonghong Lan

**Affiliations:** a Key Laboratory of Tropical Translational Medicine of Ministry of Education, School of Basic Medicine and Life Sciences, Hainan Medical University, Haikou, Hainan PR China.

**Keywords:** cervical cancer, computer image analysis, discriminant analysis, liquid-based smear, squamous cell

## Abstract

This study aimed to explore a discriminant method for cervical squamous epithelial cells based on computer image analysis to establish a foundation for artificial intelligence diagnosis of cervical cancer. A total of 1682 cells were captured from 53 Papanicolaou smears, and computer image analysis was used to test the chromatics and geometric structural parameters of the above cells. Stepwise discriminant analysis was used to establish the discriminant function, and the discriminant effects of the function were evaluated. The chromaticity and geometric features of the cell images had significantly different among low-grade cells (LGC), atypical squamous cells of undetermined significance, and high-grade cells (HGC), as well as between subclassifications within LGC and HGC (*P* < .05). Stepwise discriminant analysis was used to create functions for classifying cells into the categories LGC, atypical squamous cells of undetermined significance, and HGC, as well as subclassifications within LGC and HGC. Functions combined with chromatics and geometry features have a good discriminant effect on cervical squamous cells. The discriminant coincidence rates indicate that this method can be an appropriate reference approach for the classification and diagnosis of cervical squamous epithelial cells, and its potential applications are presented in a tentative study on automated image analysis systems for cytological fields.

## 1. Introduction

Cervical cancer is one of the leading causes of cancer-related death among women. Cervical cancer is the fourth most frequently occurring malignancy in women worldwide, resulting in an estimated 6,04,000 new cases annually, with 3,42,000 deaths worldwide in 2020.^[1,2]^ Approximately 85% of cervical cancer deaths worldwide occur in underdeveloped or developing countries, and the death rate is 18 times higher in low-income and middle-income countries than in wealthier countries.^[3,4]^ Cervical cancer is one of the most prevalent and treatable cancers in the world. Cervical cancer begins with precancerous changes and develops gradually. Thus, up to 92% of this cancer may be prevented because cell changes are detected and treated early using the Smear Test.^[5]^

Early detection is achieved by Papanicolaou (Pap) screening of cervical cells. The Pap test is the most popular and successful method for preventing cervical cancer and has been decelerated in developed countries since it started using the Pap test. The shortage of skilled cytotechnologists for screening and diagnosing Pap slides has always been a concern, driving the development of automated systems. Owing to the revolution and evolution of new technologies, enhanced power of computation, decreased cost of hardware and software, and the prevalence of the Internet, an increasing number of systems have been developed using computational algorithms for cellular image analysis.^[6,7]^ Such approaches promise to resolve the limitations of subjective analysis, especially in the fields of bioinformatics, biology, and medicine.^[8,9]^

The aim of this study was to test the morphological parameters (chromaticity and geometry parameters) of cervical squamous cells using computer image analysis, select the morphological parameters with differential values to establish the discriminant function, and use the function to diagnose and classify cervical squamous cells. This effectively avoids the traditional qualitative observation of subjectivity and non-repeatability, provides convenience for large-scale cancer screening, and improves the efficiency of cervical cancer screening.

## 2. Materials and methods

### 2.1. Acquisition and categorization of cell Images

Cytological images were captured using a high-resolution digital camera (Olympus C-5060, Japan) mounted on a microscope (Olympus BX51, Japan) and stored in digital format. Cell images were captured using magnification (400×), excluding images with extensive cellular overlapping and interference from other inflammatory cells or debris.

A total of 1682 cells were captured from 53 Pap smears, of which 1149 cells were categorized as low-grade cells (LGC), including normal squamous epithelium superficial cells (NSSC, n = 682), normal squamous epithelium intermediate cells (NSMC, n = 244), low-grade squamous intraepithelial lesion cells (LSIL, n = 223), 143 cells as atypical squamous cells of undetermined significance (ASC-US), 390 cells as high-grade cells (HGC), including normal squamous epithelium basal cells (NSBC, n = 100), immature squamous epithelium metaplastic cells (IMSMC, n = 100), atypical squamous cells that could not exclude HSIL (ASC-H, n = 88), and high-grade squamous intraepithelial lesion cells (HSIL, n = 102). In addition to these cellular images, micrometer images were captured at 400× magnification to calibrate the prototype system.

### 2.2. Test and calculation of morphological parameter

Morphological parameters of cervical squamous cell – including red (*R*), green (*G*), blue (*B*) and area (*A*), major axis ($d_{maj}$), minor axis ($d_{min}$), average diameter (*D*), perimeter (*P*) of cytoplasm and nucleus – were analyzed using image analysis software (image-pro Plus 6.0, Media Cybernetics, Tampa), and other parameters – red, green, blue primary color coefficient (*r*, *g*, *b*) and nucleocytoplasmic ratio ($R_{np}$), form irregular index and regular form factor – were calculated, the calculation formula is as follows:


$$\begin{array}{l} r=\frac{R}{R+G+B}\;\;\;\;\;\;\;\;\;\;\;\;\;\;g=\frac{G}{R+G+B}\;\;\;\;\;\;\;\;\;\;\;\;\;\;\;\;b=\frac{B}{R+G+B} \end{array}$$

$$\begin{array}{l} \;\;\;R_{np}=\frac{A_{n}}{A_{p}}\;\;\;\;\;\;\;\;\;FII=\frac{P}{\sqrt{4\pi A}}\;\;\;\;\;\;RFF=\frac{A\left[3\left(d_{maj}+d_{min}\right)-2\sqrt{d_{maj}*d_{min}}\right]}{d_{maj}*d_{min}*P} \end{array}$$

### 2.3. Discrimination step of cells

First, the morphological parameters of LGC, ASC-US, and HGC were compared to select parameters with clinical diagnostic value, and a stepwise discriminant analysis was performed to establish the discriminant function to classify LGC, ASC-US, and HGC. The morphological parameters of LGC (NSSC, NSMC, and LSIL) and HGC (NSBC, ASC-H, IMSMC, and HSIL) were analyzed according to the above steps, and the classification and diagnosis of cells were obtained.

### 2.4. Statistical analysis

Statistical analyses were performed using SPSS21.0 (IBM, Armonk). The test data were analyzed using One-Way ANOVA. Stepwise discriminant analysis was used to establish the discriminant function and evaluate the coincidence rate of the discriminant function, and *P *< .05 was considered statistically significant.

## 3. Results

**Figure 1.**
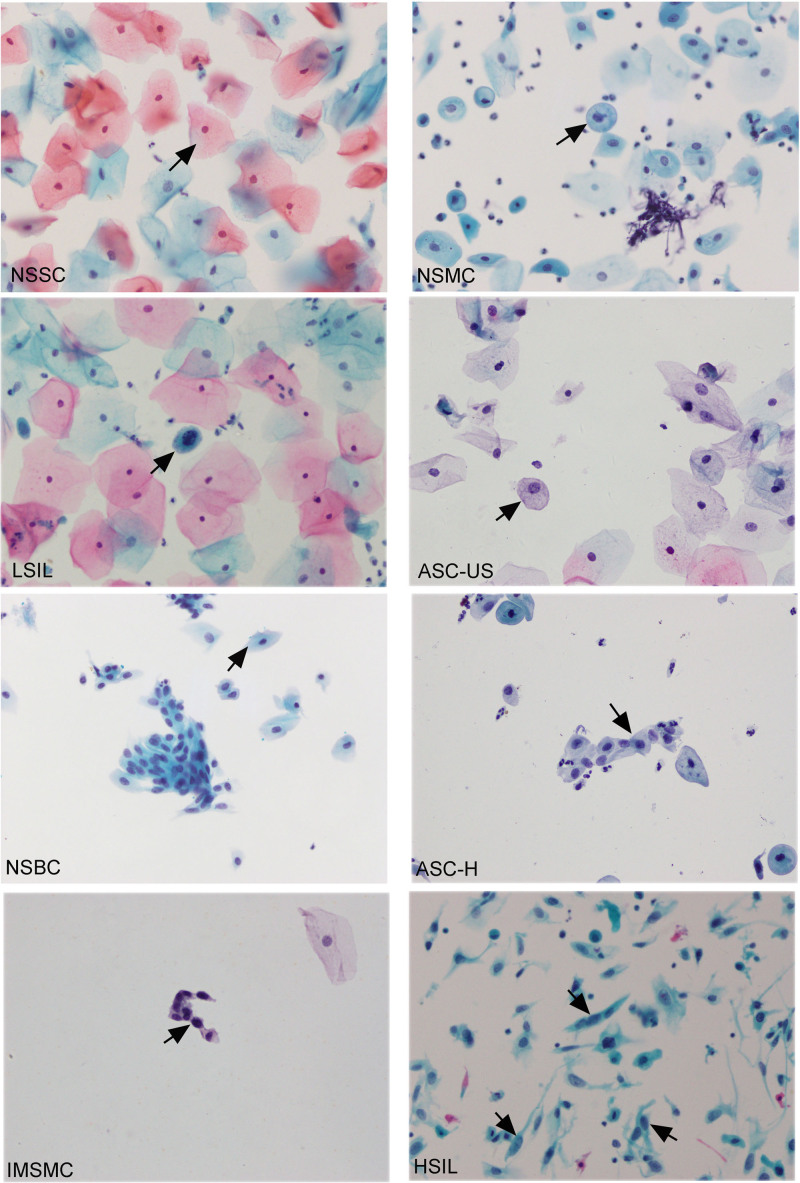
Microscope image of squamous cells (Pap staining, 400×). ASC-H = atypical squamous cells that could not exclude HSIL, ASC-US = atypical squamous cells of undetermined significance, HSIL = high-grade squamous intraepithelial lesion, IMSMC = immature squamous epithelium metaplastic cells, LSIL = low-grade squamous intraepithelial lesion cells, NSBC = normal squamous epithelium basal cells, NSMC = normal squamous epithelium intermediate cells, NSSC = normal squamous epithelium superficial cells, Pap = Papanicolaou.

Three types of squamous cells were observed in the smear images. LGC (NSSC, NSMC, and LSIL) is the largest cells and has small nuclei and rich cytoplasm that is generally light-stained. USC-US is slightly smaller in size, has less cytoplasm, and larger deeply stained nuclei. HGC (NSBC, ASC-H, IMSMC, and HSIL) with a small cell body are an immature cell type with few cytoplasms and irregular nuclei that are deeply stained, Figure 1.

### 3.1. Morphological parameters

There were significant differences in chromaticity parameter ($r_{p}$ $b_{p}$ $r_{n}$ $b_{n}$) and geometry parameter ($A_{c}$ $A_{n}$ $d_{maj,c}$ $d_{min,c}$ $d_{maj,n}$ $d_{min,n}$ $D_{c}$ $D_{n}$ $P_{c}$ $P_{n}$ $R_{np}$ $FII_{n}$ $RFF_{c}$ $RFF_{n}$) among LGC, ASC-US, HGC (*P* < .05), Table 1. There were significant differences in chromaticity parameter ($r_{p}$ $g_{p}$ $b_{p}$ $r_{n}$ $g_{n}$ $b_{n}$) and geometry parameter ($A_{c}$ $A_{n}$ $d_{maj,c}$ $d_{min,c}$ $d_{maj,n}$ $d_{min,n}$ $D_{c}$ $D_{n}$ $P_{c}$ $P_{n}$ $R_{np}$ $FII_{n}$ $RFF_{c}$ $RFF_{n}$) among NSSC, NSMC, LSIL (*P* < .05), Table 2. There were significant differences in chromaticity parameter ($r_{p}$ $g_{p}$ $b_{p}$ $r_{n}$ $g_{n}$ $b_{n}$) and geometry parameter ($A_{c}$ $A_{n}$ $d_{maj,\;c}$ $d_{min,c}$ $d_{maj,n}$ $d_{min,n}$ $D_{c}$ $D_{n}$ $P_{c}$ $P_{n}$ $R_{np}$ $FII_{n}$ $RFF_{c}$ $RFF_{n}$) among NSBC, ASC-H, IMSMC, HSIL (*P* < .05), Table 3.

**Table 1:** Comparison with morphological parameters of LGC, ASC-US and HGC.

Parameters	LGC (1149)	AUS-US (143)	HGC (390)	*F*	*P*
$r_{p}$	0.325 ± 0.063	0.284 ± 0.055	0.308 ± 0.080	29.137	.001
$g_{p}$	0.339 ± 0.047	0.338 ± 0.031	0.335 ± 0.043	1.321	.267
$b_{p}$	0.336 ± 0.088	0.378 ± 0.060	0.357 ± 0.085	21.021	.001
$r_{n}$	0.300 ± 0.075	0.255 ± 0.060	0.277 ± 0.056	35.158	.001
$g_{n}$	0.287 ± 0.089	0.303 ± 0.063	0.289 ± 0.087	2.077	.126
$b_{n}$	0.413 ± 0.095	0.442 ± 0.068	0.434 ± 0.106	10.966	.001
$A_{c}$ (μm^2^)	1345.836 ± 775.958	524.150 ± 231.013	334.133 ± 269.422	392.406	.001
$A_{n}$ (μm^2^)	93.479 ± 65.880	110.833 ± 61.081	91.900 ± 53.814	5.307	.005
$d_{maj,c}$ (μm)	46.987 ± 14.504	31.482 ± 7.181	24.566 ± 10.014	463.766	.001
$d_{min,c}$ (μm)	33.633 ± 12.315	20.758 ± 5.630	15.499 ± 7.017	444.546	.001
$d_{maj,n}$ (μm)	11.962 ± 4.068	13.455 ± 3.671	12.338 ± 3.637	9.562	.001
$d_{min,n}$ (μm)	9.134 ± 2.999	9.944 ± 2.696	8.938 ± 2.778	6.306	.002
$D_{c}$ (μm)	38.681 ± 12.847	24.590 ± 5.646	18.617 ± 7.536	496.544	.001
$D_{n}$ (μm)	10.206 ± 3.288	11.295 ± 2.916	10.229 ± 2.890	7.652	.001
$P_{c}$ (μm)	136.642 ± 42.818	89.180 ± 18.710	68.913 ± 27.859	497.527	.001
$P_{n}$ (μm)	34.387 ± 11.670	38.444 ± 10.498	35.115 ± 10.267	8.342	.001
$R_{np}$	0.186 ± 0.342	0.342 ± 0.250	0.716 ± 0.971	134.544	.001
$FII_{c}$	0.926 ± 0.033	0.924 ± 0.038	0.922 ± 0.046	2.053	.129
$FII_{n}$	0.968 ± 0.042	0.960 ± 0.034	0.955 ± 0.038	16.007	.001
$RFF_{c}$	1.115 ± 0.094	1.133 ± 0.107	1.150 ± 0.136	16.461	.001
$RFF_{n}$	1.051 ± 0.083	1.063 ± 0.047	1.074 ± 0.061	12.979	.001

ASC-US = atypical squamous cells of undetermined significance, FII = form irregular index, HGC = high-grade cells, LGC = low-grade cells, RFF = regular form factor.

**Table 2:** Comparison with morphological parameters of low-grade cell.

Parameters	NSSC (682)	NSMC (244)	LSIL (223)	*F*	*P*
$r_{p}$	0.352 ± 0.055	0.271 ± 0.044	0.301 ± 0.052	247.735	.001
$g_{p}$	0.339 ± 0.057	0.346 ± 0.030	0.330 ± 0.024	6.867	.001
$b_{p}$	0.309 ± 0.097	0.383 ± 0.053	0.369 ± 0.044	99.691	.001
$r_{n}$	0.329 ± 0.071	0.238 ± 0.049	0.275 ± 0.060	198.153	.001
$g_{n}$	0.282 ± 0.104	0.305 ± 0.062	0.285 ± 0.057	6.079	.002
$b_{n}$	0.389 ± 0.105	0.457 ± 0.074	0.440 ± 0.051	62.748	.001
$A_{c}$ (μm^2^)	1671.558 ± 676.317	646.257 ± 589.527	1115.140 ± 642.482	239.316	.001
$A_{n}$ (μm^2^)	62.687 ± 26.631	128.578 ± 91.449	149.247 ± 61.018	281.345	.001
$d_{maj,c}$ (μm)	53.062 ± 10.784	32.913 ± 14.691	43.809 ± 12.393	261.883	.001
$d_{min,c}$ (μm)	39.103 ± 9.594	21.199 ± 11.116	30.509 ± 9.748	303.641	.001
$d_{maj,n}$ (μm)	9.947 ± 2.124	14.438 ± 5.109	15.416 ± 3.462	328.668	.001
$d_{min,n}$ (μm)	7.785 ± 1.701	10.377 ± 3.795	11.903 ± 2.625	272.476	.001
$D_{c}$ (μm)	44.485 ± 9.467	25.340 ± 12.130	35.526 ± 10.414	325.356	.001
$D_{n}$ (μm)	8.609 ± 1.793	11.900 ± 4.049	13.239 ± 2.804	324.950	.001
$P_{c}$ (μm)	155.649 ± 31.235	93.324 ± 42.353	125.909 ± 35.165	304.214	.001
$P_{n}$ (μm)	28.546 ± 6.172	41.370 ± 14.478	44.610 ± 9.725	342.321	.001
$R_{np}$	0.052 ± 0.054	0.537 ± 0.591	0.214 ± 0.149	265.094	.001
$FII_{c}$	0.929 ± 0.027	0.918 ± 0.045	0.927 ± 0.031	10.102	.001
$FII_{n}$	0.977 ± 0.042	0.946 ± 0.045	0.963 ± 0.025	57.468	.001
$RFF_{c}$	1.100 ± 0.050	1.161 ± 0.171	1.110 ± 0.053	40.503	.001
$RFF_{n}$	1.038 ± 0.096	1.086 ± 0.067	1.052 ± 0.031	30.798	.001

FII = form irregular index, LSIL = low-grade squamous intraepithelial lesion cells, NSMC = normal squamous epithelium intermediate cells, NSSC = normal squamous epithelium superficial cells, RFF = regular form factor.

**Table 3:** Comparison on morphological parameters of high-grade cell.

Parameters	NSBC (100)	ASC-H (88)	IMSMC (100)	HSIL (102)	*F*	*P*
$r_{p}$	0.311 ± 0.026	0.263 ± 0.052	0.295 ± 0.053	0.355 ± 0.122	26.057	.001
$g_{p}$	0.318 ± 0.016	0.327 ± 0.026	0.341 ± 0.039	0.351 ± 0.064	13.126	.001
$b_{p}$	0.371 ± 0.028	0.409 ± 0.044	0.364 ± 0.062	0.293 ± 0.123	40.596	.001
$r_{n}$	0.285 ± 0.037	0.244 ± 0.046	0.261 ± 0.069	0.313 ± 0.043	34.176	.001
$g_{n}$	0.248 ± 0.026	0.271 ± 0.054	0.296 ± 0.070	0.336 ± 0.131	21.549	.001
$b_{n}$	0.467 ± 0.039	0.485 ± 0.043	0.443 ± 0.087	0.351 ± 0.150	40.514	.001
$A_{c}$ (μm^2^)	160.823 ± 151.219	376.845 ± 261.897	395.332 ± 209.594	407.198 ± 338.507	21.752	.001
$A_{n}$ (μm^2^)	63.636 ± 21.095	93.029 ± 59.527	92.607 ± 46.516	117.942 ± 63.773	19.645	.001
$d_{maj,c}$ (μm)	16.561 ± 5.274	25.617 ± 8.899	28.295 ± 8.180	27.850 ± 11.666	38.561	.001
$d_{min,c}$ (μm)	11.542 ± 3.854	16.786 ± 7.502	17.152 ± 6.555	16.647 ± 7.970	16.006	.001
$d_{maj,n}$ (μm)	10.301 ± 1.726	12.473 ± 4.089	12.622 ± 3.690	13.941 ± 3.671	19.822	.001
$d_{min,n}$ (μm)	7.806 ± 1.477	8.790 ± 3.160	8.945 ± 2.572	10.169 ± 3.110	13.480	.001
$D_{c}$ (μm)	13.385 ± 4.150	20.004 ± 7.744	20.833 ± 6.410	20.378 ± 8.554	25.998	.001
$D_{n}$ (μm)	8.759 ± 1.422	10.158 ± 3.282	10.319 ± 2.663	11.642 ± 3.129	19.144	.001
$P_{c}$ (μm)	47.294 ± 14.903	72.018 ± 26.807	79.196 ± 22.303	77.349 ± 31.894	35.592	.001
$P_{n}$ (μm)	29.655 ± 4.900	10.158 ± 3.282	10.319 ± 2.663	11.642 ± 3.129	18.504	.001
$R_{np}$	1.268 ± 1.365	0.452 ± 0.245	0.442 ± 0.395	0.671 ± 1.078	17.680	.001
$FII_{c}$	0.935 ± 0.030	0.934 ± 0.360	0.910 ± 0.049	0.910 ± 0.057	9.937	.001
$FII_{n}$	0.956 ± 0.034	0.960 ± 0.035	0.948 ± 0.046	0.956 ± 0.033	1.854	.137
$RFF_{c}$	1.102 ± 0.063	1.128 ± 0.083	1.190 ± 0.182	1.177 ± 0.154	9.927	.001
$RFF_{n}$	1.062 ± 0.044	1.076 ± 0.063	1.087 ± 0.082	1.070 ± 0.045	3.058	.001

ASC-H = atypical squamous cells that could not exclude HSIL, FII = form irregular index, HSIL = high-grade squamous intraepithelial lesion, IMSMC = immature squamous epithelium metaplastic cells, NSBC = normal squamous epithelium basal cells, RFF = regular form factor.

### 3.2. Stepwise discriminant analysis

The function (1) was established to classify LGC, ASC-US, HGC based on chromaticity parameter ($r_{p}$ $b_{p}$ $r_{n}$ $b_{n}$) and geometry parameter ($A_{c}$ $A_{n}$ $d_{maj,c}$ $d_{min,c}$ $d_{maj,n}$ $d_{min,n}$ $D_{c}$ $D_{n}$ $P_{c}$ $P_{n}$ $R_{np}$ $FII_{n}$ $RFF_{c}$ $RFF_{n}$). In function (1), *Y*_1_, *Y*_2_ and *Y*_3_ are the discriminant functions of LGC, ASC-US, and HGC, respectively.


$$\begin{array}{l} \left\{ \begin{array}{lllllllllllllllllll} & Y_{1}-4121.966+1656.889r_{p}+1910.036b_{p}\; & +267.662\;r_{n}+1061.112b_{n}-0.061A_{c}\; & -0.102\;A_{n}+1.867\;d_{maj,c}-2.459\;\;\;d_{min,\;c}\; & +2.101\;d_{maj,n}+67.041\;d_{min,n}\;+30.122\;D_{c}\; & -57.459\;D_{n}-7.493\;P_{c}+29.084\;R_{np}\; & +3929.452\;FII_{n}+497.049\;RFF_{c}+2099.652\;RFF_{n}\; & Y_{2}-4122.483+1657.769r_{p}+1918.251\;b_{p}\; & +273.399\;r_{n}+1067.207\;b_{n}-0.060A_{c}\; & -0.112\;A_{n}+2.007\;d_{maj,c}-2.236\;d_{min,c}\; & +2.104\;d_{maj,n}+66.853d_{min,n}+29.706\;D_{c}\; & -56.978\;D_{n}-7.523\;P_{c}+28.438\;R_{np}+3929.990FII_{n}\; & +494.618\;RFF_{c}+2098.033\;RFF_{n}\; & Y_{3}-4114.923+1659.657\;r_{p}+1909.207\;b_{p}\; & +273.762\;r_{n}+1059.352\;b_{n}-0.056A_{c}\; & -0.115\;A_{n}+1.868\;d_{maj,c}-2.349\;d_{min,c}\; & +2.425\;d_{maj,n}+67.406\;d_{min,n}+29.409\;D_{c}\; & \;-57.748\;D_{n}-7.454\;P_{c}+29.135\;R_{np}+3931.511\;FII_{n}\; & +492.400\;RFF_{c}+2100.210\;RFF_{n}\; \end{array}\right. \end{array}$$

The function (2) was established to classify NSSC, NSMC, LSIL based on chromaticity parameter ($r_{p}$ $g_{p}$ $b_{p}$ $r_{n}$ $g_{n}$ $b_{n}$) and geometry parameter ($A_{c}$ $A_{n}$ $d_{maj,c}$ $d_{min,c}$ $d_{maj,n}$ $d_{min,n}$ $D_{c}$ $D_{n}$ $P_{c}$ $P_{n}$ $R_{np}$ $FII_{n}$ $RFF_{c}$ $RFF_{n}$). In function (2), *Y*_4_, *Y*_5_ and *Y*_6_ are the discriminant functions of NSSC, NSMC, and LSIL, respectively.


$$\begin{array}{l} \left\{ \begin{array}{lllllllllllllllllllll} & Y_{4}-7071.601+1682.311r_{p}+1635.775\;b_{p}\; & +100.372\;r_{n}+933.041\;g_{n}-0.046A_{c}\; & -0.507A_{n}-95.531\;d_{maj,c}-97.041\;\;d_{min,c}\; & -6.749\;d_{maj,n}+46.755\;d_{min,n}+83.378\;D_{c}\; & -21.194\;D_{n}+34.034P_{c}+57.252\;R_{np}\; & +6016.315\;FII_{c}+3716.541\;FII_{n}+1342.620\;RFF_{c}\; & +1829.270\;RFF_{n}\; & Y_{5}-7068.932+1648.164\;r_{p}+1620.851\;b_{p}\; & +101.267\;r_{n}+929.463\;g_{n}-0.033A_{c}\; & -0.0481\;A_{n}-96.759\;d_{maj,c}-98.380\;d_{min,c}\; & -5.000\;d_{maj,n}+48.800\;d_{min,n}+83.698\;D_{c}\; & -24.617\;D_{c}+34.396P_{c}+57.308\;R_{np}\; & +6055.519\;FII_{c}+3700.700\;FII_{n}+1352.893\;RFF_{c}\; & +1825.382\;RFF_{n}\; & Y_{6}-7068.644+1690.914\;r_{p}+1641.410\;\;b_{p}\; & +95.538\;\;r_{n}+931.665\;\;g_{n}-0.037\;A_{c}\; & -0.526\;A_{n}-95.874\;d_{maj,c}-97.474\;d_{min,c}\; & -5.550\;d_{maj,n}+48.416\;d_{min,n}+83.213\;D_{c}-22.645\;D_{n}\; & +34.106P_{c}+54.993\;R_{np}+6017.458\;{FII}_{c}\; & +3715.632\;{FII}_{n}+1341.316\;{RFF}_{c}+1827.847\;{RFF}_{n}\; \end{array}\right. \end{array}$$

The function (3) was established to classify NSBC, ASC-H, IMSMC, HSIL based on chromaticity parameter ($r_{p}$ $g_{p}$ $b_{p}$ $r_{n}$ $g_{n}$ $b_{n}$) and geometry parameter ($A_{c}$ $A_{n}$ $d_{maj,c}$ $d_{min,c}$ $d_{maj,n}$ $d_{min,n}$ $D_{c}$ $D_{n}$ $P_{c}$ $P_{n}$ $R_{np}$ $FII_{n}$ $RFF_{c}$ $RFF_{n}$). In function (3), *Y*_7_, *Y*_8_, *Y*_9_ and *Y*_10_ are the discriminant functions of the NSBC, ASC-H, IMSMC, and HSIL, respectively.


$$\begin{array}{l} \left\{ \begin{array}{lllllllllllllllllllllll} & Y_{7}-10481.271+3305.315\;r_{p}+4075.893\;b_{p}\; & +436.712\;r_{n}+1903.729\;g_{n}-0.688A_{c}+3.070\;A_{n}\; & -27.767\;d_{maj,c}-47.865\;d_{min,c}-168.145\;d_{maj,n}+64.225\;d_{min,n}\; & +62.984\;D_{c}+77.840\;D_{n}+11.888\;P_{c}+45.673\;R_{np}\; & +9982.419\;FII_{c}-344.354\;RFF_{c}+8166.241\;RFF_{n}\; & Y_{8}-10577.598+3315.593\;r_{p}+4099.091\;b_{p}+425.450\;r_{n}\; & +1921.436\;g_{n}-0.700A_{c}+3.158\;A_{n}-27.720\;d_{maj,c}\; & -48.357d_{min,c}-168.374\;d_{maj,n}+64.679\;d_{min,n}\;\; & +64.684\;D_{c}+75.889\;D_{n}+11.685\;P_{c}+45.027\;R_{np}\; & +10025.957\;FII_{c}-335.785\;RFF_{c}+8199.084\;RFF_{n}\; & Y_{9}-10524.001+3301.032\;r_{p}+4070.575\;b_{p}\; & +428.167\;r_{n}+1908.840\;g_{n}-0.708A_{c}\; & +3.129A_{n}-27.567\;d_{maj,c}-48.000\;d_{min,c}\; & -168.303\;d_{maj,n}+64.302\;d_{min,n}+64.593\;D_{c}\; & +76.700\;D_{n}+11.662\;P_{c}+45.197\;R_{np}\; & +9995.757\;FII_{c}-329.598\;RFF_{c}+8179.981\;RFF_{n}\; & Y_{10}-10511.707+3302.306\;r_{p}+4065.558\;b_{p}\; & +448.292\;r_{n}+1914.442\;g_{n}-0.692A_{c}\; & +3.121\;A_{n}-27.349\;d_{maj,c}-47.660\;d_{min,c}\; & -169.300\;d_{maj,n}+62.499\;d_{min,n}+63.422\;D_{c}\; & +80.180\;D_{n}+11.635\;P_{c}+45.224\;R_{np}\; & +9991.434\;FII_{c}-332.436\;RFF_{c}+8170.679\;RFF_{n}\; \end{array}\right. \end{array}$$

### 3.3. Discriminant effects of function

The discriminant coincidence rates of function (1) for LGC, ASC-US, and HGC were 71.7, 73.4, and 64.6%, respectively, and the overall coincidence rate was 70.2% (Table 4). The discriminant coincidence rates of function (2) for the NSSC, NSMC, and LSIL were 91.5%, 70.9%, and 74.0%, respectively, and the overall coincidence rate was 83.7% (Table 5). The discriminant coincidence rates of function (3) for NSBC, ASC-H, IMSMC, and HSIL were 80.0, 60.2, 62.0, and 62.7%, respectively, and the overall coincidence rate was 66.4% (Table 6).

**Table 4:** The discriminant coincidence rate of function (1).

Original cell	Predicted cell				Accuracy (%)
	LGC	ASC-US	LGC	Total	
LGC	824	209	116	1149	71.7
ASC-US	14	105	24	143	73.4
HGC	21	117	252	390	64.6
Total	859	431	392	1682	70.2

ASC-US = atypical squamous cells of undetermined significance, HGC = high-grade cells, LGC = low-grade cells.

**Table 5:** The discriminant coincidence rate of function (2).

Original cell	Predicted cell				Accuracy (%)
	NSSC	NSMC	LSIL	Total	
NSSC	624	11	47	682	91.5
NSMC	15	173	56	244	70.9
LSIL	20	38	165	223	74.0
Total	659	222	268	1149	83.7

LSIL = low-grade squamous intraepithelial lesion cells, NSMC = normal squamous epithelium intermediate cells, NSSC = normal squamous epithelium superficial cells.

**Table 6:** The discriminant coincidence rate of function (3).

Original cell		Predicted cell				Accuracy (%)
	NSBC	ASC-H	IMSMC	HSIL	Total	
NSBC	80	7	9	4	100	80.0
ASC-H	13	53	18	4	88	60.2
IMSMC	7	17	62	14	100	62.0
HSIL	18	2	18	64	102	62.7
Total	118	79	107	86	390	66.4

ASC-H = atypical squamous cells that could not exclude HSIL, HSIL = high-grade squamous intraepithelial lesion, IMSMC = immature squamous epithelium metaplastic cells, NSBC = normal squamous epithelium basal cells.

## 4. Discussion

Cervical cancer begins in the cervix, which is the lower part of the uterus (sometimes called the uterine cervix) and connects the body of the uterus to the vagina. The endocervix is part of the cervix close to the body of the uterus, whereas the exto-cervix is part of the vagina.^[10]^ Approximately 80% to 90% of cervical cancers originate from squamous cells that cover the surface of the exto-cervix.^[11]^

In the developed world, cervical cancer screening is performed using cytological examination of cervical smears. After visualization of the cervix using a speculum, the specimen is obtained using a sampling device, usually a spatula or brush, which is rotated on the cervix. The collected material was applied to a glass slide (for conventional cytology) or the sampling device was rinsed in or left in a preservative solution (for liquid-based cytology).^[12]^ Visual interpretation of Pap smear images is tedious, time-consuming, and error-prone.^[13]^ Simultaneously, there is a shortage of skilled cytologists to screen and diagnose the sudden increase in the workload of smears,^[14]^ and many laboratories face similar challenges.^[15,16]^ Liquid-based cytology for cervical cancer screening is now more common than conventional smears, which when digitized from glass slides into whole-slide images, opens up the possibility of artificial intelligence-based automated image analysis.^[17,18]^ Conventional screening processes by cytoscreeners and cytopathologists using microscopes are limited in terms of human resources. Therefore, it is important to develop new computational techniques that can automatically and rapidly diagnose a large number of specimens without delay, which would be of great benefit to clinical laboratories and hospitals.^[19,20]^

Pathological changes in cell structure are manifested by certain morphological changes, including changes in geometric structure and color features. Our results suggest that morphological parameters tested by image analysis technology have diagnostic and classification values, and discriminant analysis was performed on morphological parameters that received good discriminant coincidence rates in the diagnosis and classification of cervical squamous cells. Computer image analysis technology combined with automatic cell recognition systems can be developed and popularized, which can alleviate the lack of cytologists and avoid the subjectivity of diagnosis to a certain extent. Overall, we believe that this technology will significantly contribute to cervical cancer screening. This approach can also assist in cellular classification in other cytological fields. Additional problems related to interobserver variation and ambiguous diagnosis can be resolved through a combination of clinical, cytomorphological, and morphometric variables.

## Author contributions

**Conceptualization:** Haiyan Niu, Yonghong Lan.

**Data curation:** Haiyan Niu, Jing Zheng, Na Xie, Chaona Jiang, Yonghong Lan.

**Formal analysis:** Chaona Jiang, Yonghong Lan.

**Methodology:** Haiyan Niu, Jing Zheng, Na Xie, Chaona Jiang, Yonghong Lan.

**Resources:** Haiyan Niu, Jing Zheng, Na Xie.

**Software:** Chaona Jiang, Yonghong Lan.

**Writing** – **original draft:** Yonghong Lan.

**Writing** – **review & editing:** Haiyan Niu.
